# Draft Genome Sequence of *Flavobacterium succinicans* Strain DD5b

**DOI:** 10.1128/genomeA.01492-16

**Published:** 2017-01-12

**Authors:** Anja Poehlein, Hristo Najdenski, Diliana D. Simeonova

**Affiliations:** a Genomic and Applied Microbiology and Goettingen Genomics Laboratory, Georg-August University Göttingen, Göttingen, Germany; b Laboratory of Zoonoses and Bacterial Virulence, Department of Infectious Microbiology, Institute of Microbiology, Bulgarian Academy of Sciences, Sofia, Bulgaria

## Abstract

We present the first 3.315-Mbp assembled draft genome sequence of *Flavobacterium succinicans* strain DD5b. This bacterium is a phosphite-assimilating representative of the genus *Flavobacterium* isolated from guts of the zooplankton *Daphnia magna*.

## GENOME ANNOUNCEMENT

*Flavobacterium succinicans* strain DD5b was isolated during a study related to diversity of bacterial communities in *Daphnia magna* guts (1, 2).

*Flavobacterium* spp. have been found in soil, fresh and marine waters, sediments, microbial mats, and glaciers (3–11). Some are fish pathogens causing bacterial gill disease, cold-water disease, or *columnaris* disease (12–15). The first *F. succinicans* strain to be isolated was from diseased fish (15, 16).

The ability of strain DD5b to assimilate phosphite as a single P source led us to investigate its genome and metabolism.

Chromosomal DNA of *F. succinicans* DD5b was isolated with a MasterPure DNA purification kit (Epicentre, Madison, WI, USA) and used to generate Illumina shotgun sequencing libraries. Sequencing was performed with a Genome Analyser II system per manufacturer instructions (Illumina, San Diego, CA, USA), resulting in 11,869,420 paired-end reads (112 bp). They were trimmed using Trimmomatic 0.32 (17). The *de novo* assembly was performed with the SPAdes genome assembler software, version 3.6.2 (18), using 4,262,625 of the reads. The assembly resulted in 74 contigs (>500 bp) and an average coverage of 126-fold. The genome of *F. succinicans* DD5b consists of a chromosome with a size of 3.315 Mb with overall G+C content of 35.18%. Automatic gene prediction was done with the software Prodigal (19). Genes coding for rRNA and tRNA were identified using RNAmmer and tRNAscan (20, 21), respectively. The Integrated Microbial Genomes–Expert Review (IMG-ER) system (22) was used for automatic annotation. The genome was manually curated using the Swiss-Prot, TrEMBL, and InterPro databases (23). This genome harbors three rRNA genes, 34 tRNA genes, 1,841 protein-coding genes with predicted functions, and 972 genes coding hypothetical proteins.

The strain is a chemoheterotroph. However, the genome contains structural elements of photosystem I, suggesting utilization of light as an energy source in an anoxygenic photoheterotrophy with concomitant mixotrophic CO_2_ fixation.

A complete copy of the cytochrome *cbb*_3_ complex and its oxidase, required for the successful colonization of anoxic tissues from different pathogens, were found as well (24, 25).

The genome of *F. succinicans* DD5b contains a two-component signal transduction pathway, encoded by *phoPR*. Phosphate requirements are covered through the low (Pit, *pitA*) and the high (Pst) affinity systems. Phosphate-selective porins and phosphate ester uptake proteins (PTS) are missing. Under phosphate starvation the strain assimilates phosphite through an alkaline phosphatase homolog *phoA*, (26), involved in the phosphate esters uptake. Other genes involved in the oxidation of inorganic phosphonates (*ptx* or *htx*) were missing. Strain DD5b assimilates phosphonoacetate as single P or C source. The phosphonoacetate hydrolase *phnA* gene was present in a single copy; however, a complete 2-aminoethyl phosphonate pathway was not identified. In nature, the biosource of phosphonoacetate is an intermediate in one of the three 2-AEP degradation pathways. Neither a carbon-phosphorus lyase complex nor a phosphonopyruvate hydrolase (*pphA*) were present in the genome.

### Accession number(s).

This whole-genome shotgun project has been deposited at DDBJ/EMBL/GenBank under the accession JMTM00000000. The version described in this paper is JMTM01000000.
